# Comparative metabolomics reveals differences in primary and secondary metabolites between “Shixia” and “Chuliang” longan (*Dimocarpus longan* Lour.) pulp

**DOI:** 10.1002/fsn3.2552

**Published:** 2021-08-28

**Authors:** Tingting Lai, Liang Shuai, Dongmei Han, Ziying Lai, Xinxin Du, Xiaomeng Guo, Wenshun Hu, Zhenxian Wu, Tao Luo

**Affiliations:** ^1^ College of Horticulture South China Agricultural University/Guangdong Provincial Key Laboratory of Postharvest Science of Fruits and Vegetables/Engineering Research Center of Southern Horticultural Products Preservation Ministry of Education Guangzhou China; ^2^ College of Food and Biological Engineering/Institute of Food Science and Engineering Technology Hezhou University Hezhou China; ^3^ Institute of Fruit Tree Research Guangdong Academy of Agricultural Sciences/Key Laboratory of South Subtropical Fruit Biology and Genetic Resource Utilization Ministry of Agriculture Guangzhou China; ^4^ Fruit Research Institute Fujian Academy of Agricultural Sciences Fuzhou China; ^5^ Key Laboratory of Biology and Genetic Improvement of Horticultural Crops (South China) Ministry of Agriculture and Rural Affairs/Guangdong Litchi Engineering Research Center Guangzhou China

**Keywords:** “Chuliang” longan, differentially accumulated metabolites, longan (*Dimocarpus longan* Lour.) pulp, “Shixia” longan, variable importance in projection (VIP) value, widely targeted metabolome

## Abstract

Longan was a characteristic fruit for both medicine and food in China, which was rich in primary and secondary metabolites. Comprehensive high‐throughput identification and comparison of metabolites in longan pulp among different varieties were still lacked. “Shixia” (SX) and “Chuliang” (CL) were the biggest major cultivars of longan in China. In this study, the content of total soluble solid, total flavonoid, and total phenolics indicated the difference of sweetness and bioactive compound content between the SX and CL pulp. Through a widely targeted metabolome, a total of 514 metabolites were identified and categorized into 23 groups mainly including flavonoids, amino acids & derivatives, lipids, phenolic acids, nucleotides & derivatives, alkaloids, organic acids and sugars & derivatives. A total of 89 metabolites with significantly differential accumulation (variable importance in projection (VIP) value ≧1, *p*‐value <.05) over 1.2 fold were found between SX and CL, which were mainly enriched into pathways including flavone and flavonol biosynthesis, glycolysis/gluconeogenesis, and arginine and proline metabolism. Higher leveled hexose and hexose‐phosphate (i.e., β‐D‐glucose, D(+)‐glucose, glucose‐1‐phosphate and glucose‐6‐phosphate), dominant organic acids (i.e., citric acid, succinic acid, D‐malic acid, and citramalate), and essential amino acids (L‐threonine, L‐valine, L‐isoleucine, L‐leucine, L‐phenylalanine and L‐lysine) in SX pulp might be contributed to the taste and flavor difference between SX and CL. Moreover, the greatly differential accumulated secondary metabolites especially flavonoids and phenolic acids might result in different medicinal and nutritional characteristic between SX and CL. In conclusion, this study provided a systemic metabolic basis for understanding the nutritional differences between SX and CL and would help deepen the molecular biology and pharmacology research on characteristic metabolites in longan pulp.

## Graphical abstract

A total of 514 metabolites were identified in SX and CL longan pulp and categorized into 23 groups by a widely targeted metabolome. A total of 89 significantly differential accumulated metabolites were found between SX and CL, which were mainly enriched into pathways including flavone and flavonol biosynthesis, glycolysis/gluconeogenesis, arginine, and proline metabolism. The greatly differential accumulated secondary metabolites especially flavonoids and phenolic acids might result in different medicinal and nutritional characteristic between SX and CL.

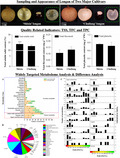

## 1 INTRODUCTION

Longan (*Dimocarpus longan* Lour.), which was originated in China and belongs to the Longan genus of *Sapindaceae*, is a famous tropical and subtropical fruit (Wu et al., 2007). As an edible fruit and traditional Chinese medicine, longan has been consumed for thousands of years (Zhang et al., 2020). Due to the high leveled primary metabolites including sugars (Chen et al., 2015; Shuai et al., 2016), organic acids (Hu et al., 2006) and amino acids (Dai et al., 2010) as well as abundant secondary metabolites containing polyphenols, flavonoids, alkaloids (Tang et al., 2019), polysaccharides (Yang et al., 2009), vitamins, nucleotides (or nucleosides) (Xiao et al., 2007), tannins, proanthocyanidins and other bioactive compounds (Sheng and Wang, 2010), the longan pulp has been used as a traditional Chinese medicine for a long history to promote blood metabolism, soothe nerves, relieve insomnia, prevent amnesia, extend longevity, cure neural pain and swelling, treat palpitation and serve as anti‐hyperglycemic agents in Asian countries (Li et al., 2015; Park et al., 2010; Yang et al., 2008; Yi et al., 2012; Zhang et al., 2020; Zhu et al., 2013).

In recent years, studies on metabolites in longan fruits were emerged and increased. Hu et al. (2006) compared the fruit qualities and analyzed the sugars and organic acids by high‐performance liquid chromatography (HPLC) in longan fruits of 12 cultivars and found the significant difference of fruit qualities, soluble solid content and related indexes among cultivars. Chen et al. (2015) analyzed the soluble sugars in the mature fruits of 63 longan germplasms and found that the main sugars including sucrose, fructose, and glucose varied among cultivars. Luo et al. (2018) found that total soluble solid (TSS) content, sugar components, and sucrose/hexose ratio in the longan pulp were different among cultivars. The difference in soluble acid invertase activities might result in the varied hexose/sucrose ratio in longan fruits among cultivars. Dai et al. (2010) determined the content of amino acids in the pulp of 18 longan varieties using HPLC and found the significant differences in amino acid content among varieties. The content of glutamic acid showed the greatest difference among longan varieties (Dai et al., 2010). Zhang et al. (2018) found that the content of phenolic acids and flavonoids as well as antioxidant capacities in the longan pulp varied among 24 cultivars, and in further suggested that the content of free and combined phenolic acids showed significantly positive correlation with the antioxidant activities (Zhang et al., 2018). However, the above‐mentioned literatures were mostly concentrated on analyzing a certain class of metabolites or small scale of metabolites and their physicochemical properties in longan fruits (Sheng and Wang, 2010; Zhang et al., 2020). High‐throughput identification and comparison of the metabolic profiles of longan pulp among different varieties were still lacked.

In the last two decades, the metabolomics based on liquid chromatography‐mass spectrometry (LC‐MS) has been widely applied in metabolome analysis on many fruit crops such as citrus (Wang et al., 2016), fig (Wang et al., 2017), tomato (Zhu et al., 2018), strawberry (*Fragaria × ananassa*) (Paolo et al., 2018), apple (Xu et al., 2020), and litchi (Guo et al., 2019). In this study, the pulp of “Shixia” and “Chuliang” longan were used as materials, and a variety of primary and secondary compounds such as sugar, organic acids, amino acids, alcohols, flavonoids, phenolic acids, nucleotides, anthocyanins, and alkaloids were determined by ultra‐high performance liquid chromatography‐mass spectrometry (UPLC/MS/MS). We screened out obviously different metabolites accumulated in the maturation process by OPLS‐DA and then annotated them using KEGG and enrichment analysis. The results revealed the difference of metabolites in the flesh of the two cultivars, which provided a theoretical basis for the evaluation of their quality and nutritional differences, and were helpful to promote the breeding of new cultivars of high quality.

## 2 MATERIALS AND METHODS

### 2.1 Longan fruits

The “Shixia” (SX) and “Chuliang” (CL) longan fruits with commercial maturity were harvested from the same orchard of Institute of Fruit Tree Research in Guangdong Academy of Agricultural Sciences on August 2, 2018, and August 7, 2018, respectively. The harvested fruits were immediately transported to the laboratory. More than 200 “Shixia” and “Chuliang” longan fruits with no disease, no damage and uniform size were selected for sampling. After the removal of seeds, the pulp of fruit was immediately frozen in liquid nitrogen and stored at −80°C until be used.

### 2.2 Chemicals

Acetic acid, methanol, and acetonitrile were HPLC degrade (Merck & Co., Inc). Ultra‐pure water was prepared by distilled water through a Milli‐Q A10 system (Millipore). Ethanol, Folin–Ciocalteau reagent, Na_2_CO_3_, gallic acid, sodium nitrite, aluminum nitrate, sodium hydroxide, rutin, and gallic acid were all analytical reagents and supplied by Sinopharm Chemical Reagent Co., Ltd.

### 2.3 Determinations of the content of total soluble solid, total phenolic and flavonoid

The pulp was separated and used for juicing and determination of the total soluble solid (TSS) content using a Brix refractometer (PAL‐1, ATAGO Co., Ltd.), and the assay was subjected to three repeats.

The ethanolic extract used for determination of total phenolic content and total flavonoid content were prepared according to our previously reported method (Shuai et al., 2021). In brief, 3 g powder of sample, ground by liquid nitrogen, was added into 3 ml 80% ethanol and extracted under ultrasonication for 30 min (with an ice bath for cooling). After a centrifugation at 5000 g for 5 min, the supernatant was transferred into a 10 ml volumetric flask. The residue was then extracted twice with 3 ml 80% ethanol as described above. The combined ethanolic extract in volumetric flask was adjusted to 10 ml using 80% ethanol and stored at −20°C for determining total phenolic content and total flavonoid content.

The total phenolic content (TPC) was determined by the Folin–Ciocalteu method (Bonilla et al., 2003) and the steps according to our previously reported method (Shuai et al., 2021). The total phenolic content was calculated according to the standard curve and expressed as mg gallic acid equivalent (GAE)/g fresh weight (FW). The assay was subjected to three repeats.

The total flavonoid content (TFC) was measured using a modified colorimetric method (Jia et al., 1999; Liu et al., 2008) and the steps according to our previously reported method (Shuai et al., 2021). The absorbance of the mixture at 510 nm was measured immediately in comparison with a standard curve prepared by rutin. The flavonoid content was expressed as mg rutin equivalent (RE)/g FW.

### 2.4 Widely targeted metabolomic analysis

#### 2.4.1 Sample extraction

The pulp was frozen by liquid nitrogen and crushed using a mixer mill (MM 400; Retsch) with a zirconia bead for 1.5 min at 30 Hz. Sample powder (100 mg) was added to 1.0 ml 70% aqueous methanol and extracted overnight at 4°C. For increasing the extraction efficiency, each sample was vortexed for three times during the period. After a centrifugation at 10,000 g 10 min, the supernatant was collected, filtered using a Carbon‐GCB SPE Cartridge (250 mg, 3 ml, CNWBOND, ANPEL) and each sample was filtrated (SCAA‐104, 0.22 μm pore size; ANPEL, http://www.anpel.com.cn/) before LC‐MS analysis.

#### 2.4.2 Ultra‐high performance liquid chromatography Separation

Sample (2 μl) was injected and analyzed using an ultra‐performance liquid chromatography (Shim‐pack UFLC CBM30A system, SHIMADZU) coupled with tandem ESI‐MS/MS (6500 Q‐TRAP, Applied Biosystems). The UPLC conditions were performed according to the previous reported method (Shuai et al., 2021): chromatographic column: ACQUITY UPLC HSS T3 (C_18_, 100 × 2.1 mm i.d., 1.8 µm, Waters); mobile phase A: ultrapure water containing 0.04% acetic acid, B: acetonitrile containing 0.04% acetic acid; elution steps: min (A, %): 0 (95%) →11.0 (5%) → 12 (5%) → 12.1 (95%) → 15 (95%); flow rate: 0.40 ml/min; column temperature: 40°C. The effluent was alternatively connected to the ESI‐triple quadrupole‐linear ion trap (Q‐TRAP)‐MS.

#### 2.4.3 ESI‐Q TRAP‐MS/MS

Widely targeted metabolites were analyzed by LIT and triple quadrupole (QQQ) scans using a triple quadrupole‐linear ion trap mass spectrometer (Applied Biosystems 6500 QTRAP) (Chen et al., 2013). The MS/MS system was equipped with an ESI Turbo Ion‐Spray interface and controlled by Analyst 1.6.3 software (AB Sciex). The parameters for operating ESI source were set as follows: ion source, turbo spray; source temperature 500°C; ion spray voltage (IS) 5500 V; ion source gas I (GSI), gas II (GSII), curtain gas (CUR) were set at 55, 60, and 25.0 psi, respectively; high collision gas (CAD). Instrument tuning and mass calibration were performed with 10 and 100 μM polypropylene glycol solutions in QQQ and LIT modes, respectively. QQQ scans were acquired as MRM experiments with collision nitrogen gas set to 5 psi. DP and CE for individual MRM transitions was done with further DP and CE optimization. A specific set of MRM transitions were monitored for each period according to the metabolites eluted within this period.

#### 2.4.4 Identification and quantitative analysis of metabolites

After the isotope signal and the repetitive signal were removed, the metabolites were qualitative by the secondary spectral information based on the public metabolite database (e.g., MassBank, KNApSAcK...) and the self‐built database MetWare database (from Metware Biotechnology Co., Ltd.).

Multiple reaction monitoring (MRM) of triple quadrupole mass spectrometry was employed to quantify each metabolite: only the precursor ions of the target substance were screened and the ionized in the collision cell to break and form fragment ions. The precursor ions and the characteristic fragment ions were selected by triple quadrupole filtration to make more accurate and repeatable quantitative results (Fraga et al., 2010). After the integration and correction of chromatographic peaks using MultiaQuant software 3.0.3 and performed on each mass spectrometry files, the relative content of the corresponding substance (area of each chromatographic peak) was calculated.

#### 2.4.5 Orthogonal partial least squares discriminant analysis and screening of differential accumulated metabolites

The metabolites that were not detected in more than two repeats of either “Shixia” or “Chuliang” were eliminated. The rest metabolites were used for orthogonal partial least squares discriminant analysis (OPLS‐DA) (Eriksson et al., 2006). OPLS‐DA was performed to eliminate the factors unrelated to sample grouping, the errors between samples and other random errors and was usually used to maximize the differences between groups. Theoretically, the more reliable model was characterized by the values of R^2^Y and Q^2^Y closer to 1. A model was generally considered as reliable if the values of R2Y and Q2Y were greater than 0.5 and the difference between them was less than 0.3. The metabolites with variable importance in projection (VIP) value ≧1, *p*‐value (SX versus CL, *t*‐test) <.05 and |log_1.2_ (SX/CL)|≧1 were identified as differential accumulated metabolites (DAMs).

#### 2.4.6 KEGG enrichment of DAMs

The cpd_id of each identified metabolite was searched in the KEGG COMPOUND database (https://www.kegg.jp/kegg/compound/) and manually examined by its exact mass. The KEGG enrichment analysis of DAMs, upregulated and downregulated DAMs were performed by the web tools: Metabolites Biological Role (MBROLE) 2.0 (López‐Ibáñez et al., 2016).

### 2.5 Statistical analysis

The variance of data was analyzed using spss software package release 18.0 (SPSS Inc). Multiple comparisons were performed by one‐way ANOVA based on Duncan's multiple range tests, while paired‐samples *t*‐tests were performed to test the statistical significance between two samples.

## 3 RESULTS

### 3.1 The content of total soluble solid, total flavonoid and total phenolics

**FIGURE 1:**
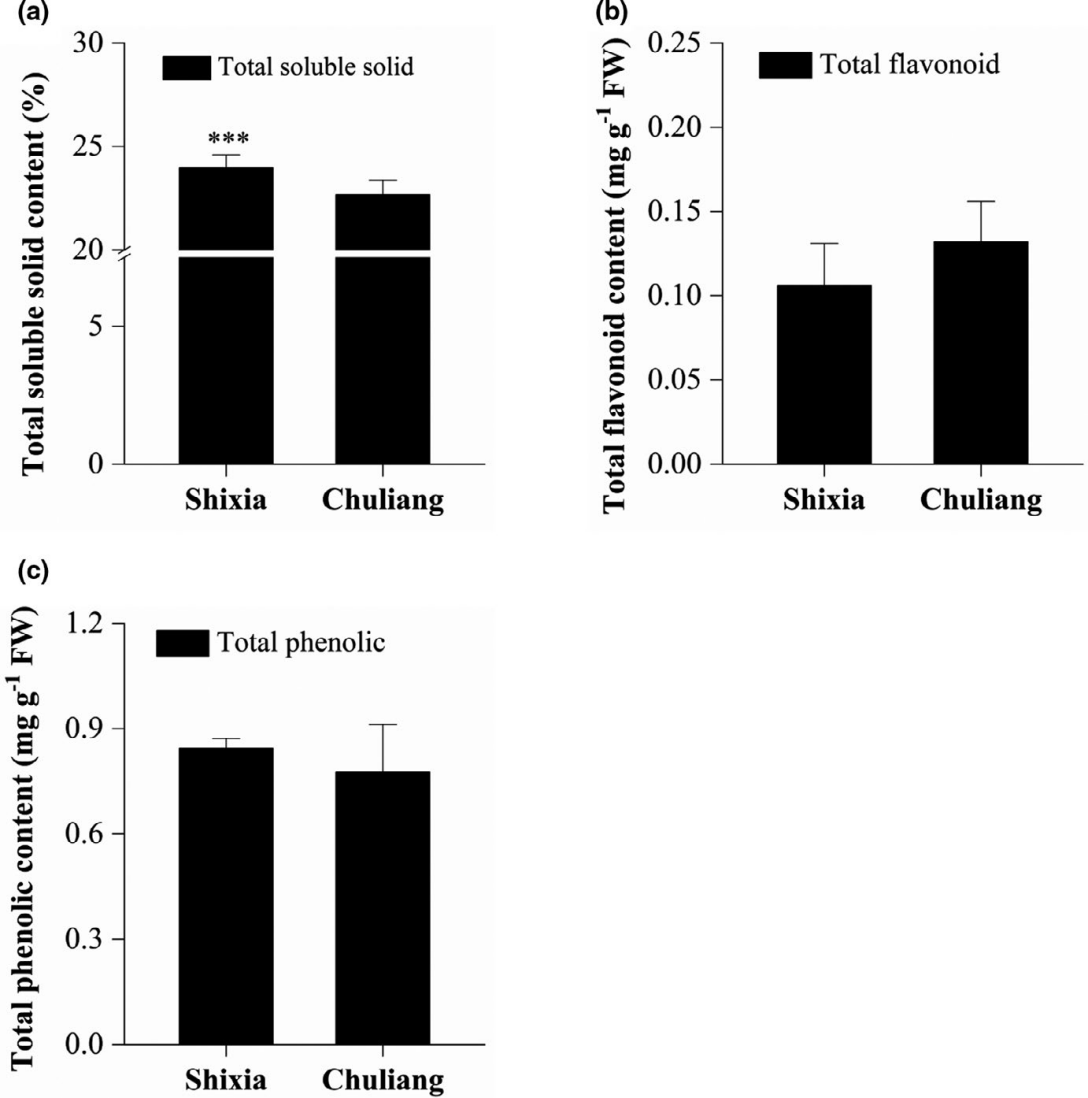
The content of total soluble solid (a), total flavonoid (b) and total phenolic (c). Note: ***p‐value <.001, two paired *t*‐test

An noteworthy significant difference was found between the TSS content of the SX longan pulp (23.98%) and that of CL longan pulp (22.67%; Figure 1a). This result was consistent with our previous result that the components of TSS as well as sucrose, glucose, and fructose in the mature longan pulp varied among cultivars and showed a significant difference between SX and CL (Luo et al., 2018). The total content of flavonoid and phenolic acids, the most abundant secondary metabolites in longan fruit, was also determined and compared between SX and CL longan pulp. The total flavonoid content in mature SX and CL longan pulp was 0.106 and 0.132 mg/g FW, respectively, while the total phenolic content in mature SX and CL longan pulp was 0.844 and 0.776 mg/g FW, respectively (Figure 1b,c). It was worthy to note that the high standard deviation and insignificant difference of total flavonoid and total phenolic content between SX and CL pulp might be resulted from the poor uniformity among individual longan fruits or biological repeats of samples. However, the above results definitely indicated the differences of sweetness and bioactive compounds content between the SX and CL pulp.

### 3.2 Identification, quantification, and classification of metabolites detected in mature SX and CL longan pulp

**FIGURE 2:**
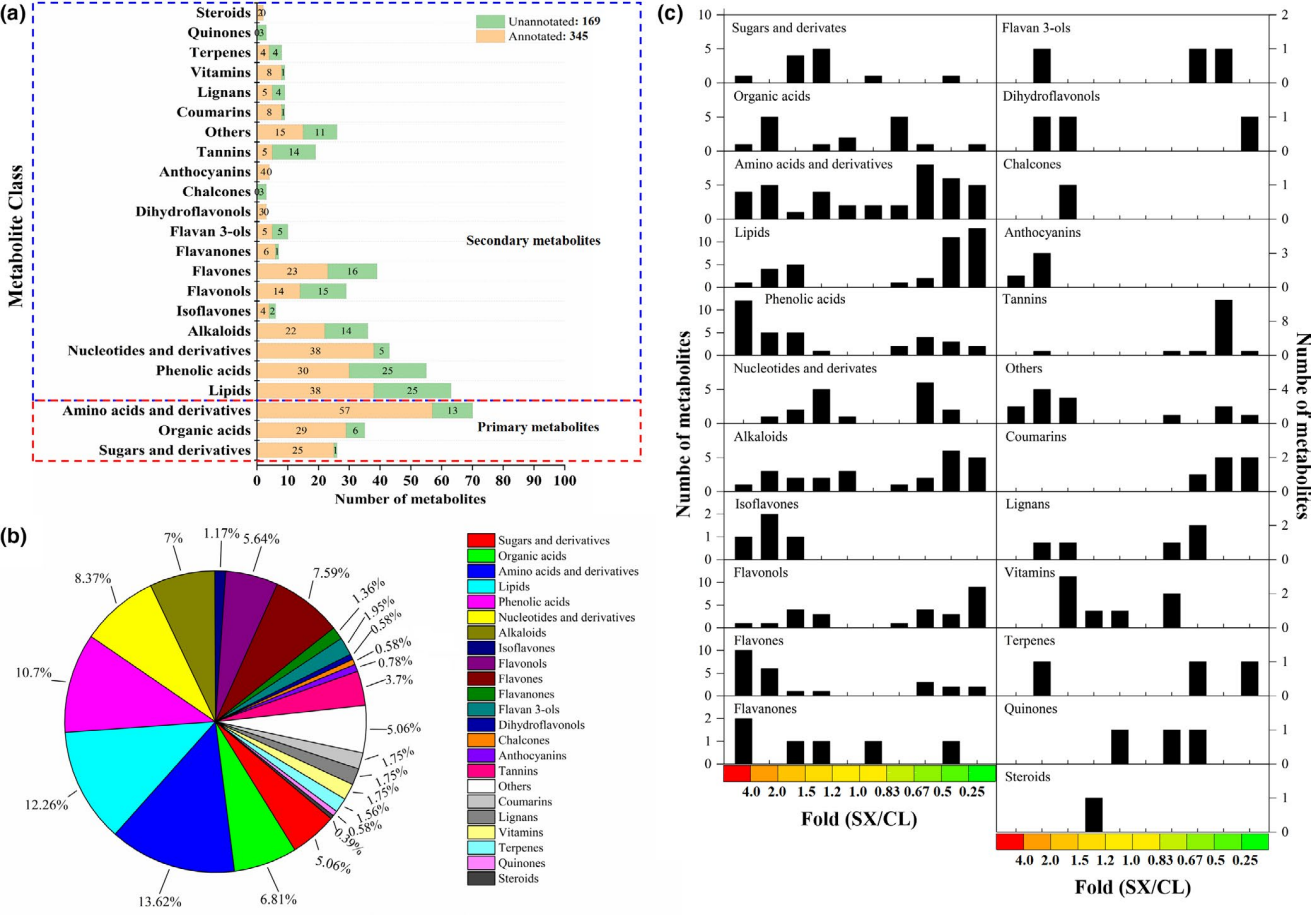
Statistics of the metabolites in “Shixia” and “Chuliang” longan pulp. (a) Statistics of the detected and annotated metabolites in each class; (b) Percentage of the metabolites in each class; (c) Statistics of metabolites in each class with reliable differences between “Shixia” and “Chuliang” longan pulp (*p*‐value <.05, two paired *t*‐test)

In order to make a systematic metabolic comparing between SX and CL longan pulp, a HPLC‐ESI‐triple quadrupole‐linear ion trap (Q‐TRAP)‐MS analysis was used to identify and quantify the metabolites. In total, 514 metabolites categorized into 23 groups were detected in both of the pulp samples. Among these metabolites, 345 metabolites were annotated with cpd_id in KEEG COMPOUND database but 169 metabolites were not annotated with any cpd_id (Figure 2a).

The largest group of secondary metabolites identified in the pulp was flavonoid, containing 97 metabolites and accounting for 18.87% of the detected metabolites (Figure 2a,b). The detected flavonoids were comprised of isoflavones (4 annotated, 2 unannotated), flavonols (14 annotated, 15 unannotated), flavones (23 annotated, 16 unannotated), flavanones (6 annotated, 1 unannotated), flavan 3‐ols (5 annotated, 5 unannotated), dihydroflavonols (3 annotated), and 3 chalcones (3 unannotated).

Moreover, 63 lipids (accounting for 12.26% of the detected metabolites, 38 annotated and 25 unannotated), 55 phenolic acids (10.70%, 30 annotated and 25 unannotated), 43 nucleotides & derivatives (8.37%, 38 annotated and 5 unannotated), 36 alkaloids (7%, 22 annotated and 14 unannotated), 19 tannins (3.7%, 5 annotated and 14 unannotated), 9 coumarins (1.75%, 8 annotated and 1 unannotated), 9 lignans (1.75%, 5 annotated and 4 unannotated), 9 vitamins (1.75%, 8 annotated and 1 unannotated), 8 terpenes (1.56%, 4 annotated and 4 unannotated), 4 anthocyanins (0.78%, 4 annotated), 3 quinones (0.58%, 3 unannotated), and 2 steroids (0.39%, 2 annotated) were detected in pulp (Figure 2a,b). In further, 26 other metabolites accounting for 5.06% of the detected metabolites (15 annotated and 11 unannotated) such as glycosides, stilbene, and xanthone were detected in longan pulp (Figure 2a,b). The largest group of primary metabolites identified in the pulp was amino acids, which was consisted of 70 amino acids and derivatives (57 annotated and 13 unannotated) accounting for 13.62% of the detected metabolites. In addition, 35 organic acids (6.81%, 29 annotated and 6 unannotated) and 26 sugars & derivatives (5.06%, 25 annotated and 1 unannotated) were detected in longan pulp (Figure 2a,b).

The numbers of upregulated and downregulated metabolites (SX versus. CL, *p*‐value <.05) in each class were displayed in a histogram (Figure 2c). All of the differently accumulated isoflavone, chalcones, anthocyanins, and steroids were found to show higher level in SX longan pulp, while all of the differently accumulated coumarins show higher level in CL longan pulp. Most of the differently accumulated sugars & derivatives, phenolic acids, flavones, flavanones, dihydroflavonols, vitamins, and other metabolites show higher level in SX longan pulp. However, most of the amino acids & derivatives, lipids, flavonols, flavan 3‐ols, tannins, terpenes, and quinones showed lower level in SX longan pulp. About half of organic acids, alkaloids, nucleotides & derivatives, flavanols, dihydroflavonols and lignans showed higher accumulation in SX while the other half of the organic acids, nucleotides & derivatives, alkaloids, and lignans showed lower accumulation in SX pulp (Figure 2c).

### 3.3 Significantly differently accumulated metabolites between mature “Shixia” and “Chuliang” pulp

**FIGURE 3:**
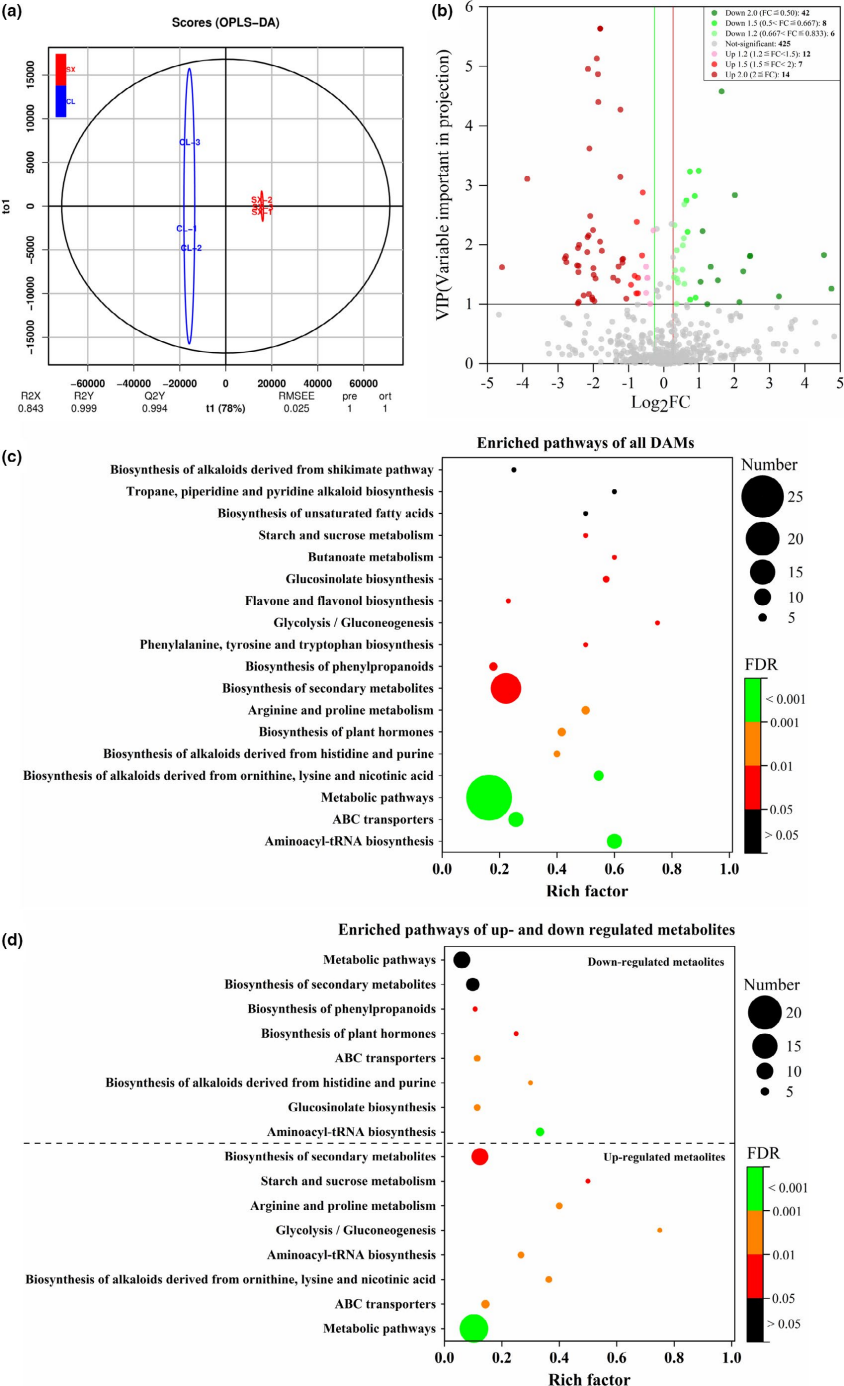
Score OPLS‐DA plot (a), volcano plot map (b), KEGG enrichment analysis of all DAMs (c) and upregulated and downregulated DAMs (d) in “Shixia” longan

Based on the OPLS‐DA analysis and significance test, the metabolites with variable importance in projection (VIP) value ≥1 and 1.2 fold‐change (SX versus CL, *t*‐test, *p*‐value <.05) were identified as the significantly differently accumulated metabolites (DAMs) between SX and CL longan pulp at mature stage. Three biological repeats of SX samples were completely separated from those of CL samples by the OPLS‐DA analysis (Figure 3a). The evaluation indexes of the model were R^2^Y(cum) = 0.999 and Q^2^Y(cum) = 0.994, which indicated the good reliability and predictability of the model. In total, 89 DAMs containing 33 upregulated metabolites and 56 downregulated metabolites were found between “Shixia” and “Chuliang” longan pulp, while 425 metabolites showed no significant difference. Among the upregulated metabolites, 14, 7, and 12 metabolites showed fold change (FC, SX versus CL) ≧ 2.0, 2.0 > FC ≧ 1.5, and 1.5 > FC ≧ 1.2, respectively. Among the downregulated metabolites, 42, 8, and 6 DAMs showed FC ≦ 0.5, 0.5 < FC ≦ 0.667, and 0.667 < FC ≦0.833, respectively (Figure 3b).

The 89 DAMs were composed of 20 flavonoid, 20 lipids, 17 amino acids, 8 alkaloids, 6 organic acids, 4 phenolic acids, 3 sugars, 3 nucleotides, 3 tannins, 1 vitamins, 1 quinones, and 3 others (Tables 1, 2, 3). Among the 26 DAMs of primary metabolites, upregulation of 7 amino acids (mainly glutamic acid, arginine, lysine, and proline), 4 organic acids (citric acid, 3‐hydroxybutyrate, citramalate, and kinic acid) and 3 sugars (glucose and glucose‐1‐phosphate) while downregulation of 10 amino acids (mainly tryptophan, phenylalanine, leucine, isoleucine, histidine...) and 2 organic acids (malic acid and 4‐guanidinobutyric acid) were observed in SX longan pulp (Table 1).

**TABLE 1:** Differentially accumulated primary metabolites in SX longan pulp compared with CL

Class	Compounds	cpd_id	Abundance (peak area)		FC	FDR	Trend
			SX	CL			↑ ↓
Amino acid & derivatives	L‐Glutamic acid	C00025	4.98 × 10^7^	4.05 × 10^7^	1.23	2.50E^−1^	7↑
	Glutamic acid	C00302	4.22 × 10^7^	3.28 × 10^7^	1.29	4.62E^−1^	
	L‐Arginine	C00062	1.07 × 10^7^	5.02 × 10^6^	2.14	1.82E^−2^	
	L‐Lysine	C00047	8.94 × 10^6^	6.20 × 10^6^	1.44	2.65E^−3^	
	Proline	C16435	4.36 × 10^6^	8.00 × 10^5^	5.45	6.62E^−4^	
	L‐Proline	C00148	4.33 × 10^6^	7.98 × 10^5^	5.42	1.07E^−3^	
	LL−2,6‐Diaminopimelic acid	C00666	1.53 × 10^6^	3.46 × 10^5^	4.41	3.55E^−3^	
	L‐Tryptophan	C00078	1.50 × 10^7^	3.52 × 10^7^	0.43	3.55E^−3^	10↓
	Phenylalanine	C02057	1.39 × 10^7^	4.86 × 10^7^	0.29	1.59E^−3^	
	L‐Phenylalanine	C00079	1.05 × 10^7^	3.92 × 10^7^	0.27	1.45E^−3^	
	Tryptophan	C00806	8.22 × 10^6^	1.93 × 10^7^	0.43	6.65E^−3^	
	L‐Leucine	C00123	2.32 × 10^6^	3.86 × 10^6^	0.60	3.16E^−3^	
	L‐Isoleucine	C00407	2.12 × 10^6^	3.65 × 10^6^	0.58	7.77E^−4^	
	L‐Norleucine	C01933	2.09 × 10^6^	3.99 × 10^6^	0.52	3.67E^−4^	
	L‐Histidine	C00135	1.83 × 10^6^	7.37 × 10^6^	0.25	1.59E^−3^	
	D‐Phenylalanine	C02265	4.47 × 10^5^	1.95 × 10^6^	0.23	1.35E^−3^	
	S‐(5′‐Adenosy)‐L‐homocysteine	C00021	2.52 × 10^5^	1.36 × 10^6^	0.18	7.77E^−4^	
Organic acids	Citric Acid	C00158	3.54 × 10^7^	1.14 × 10^7^	3.11	1.53E^−2^	4↑
	3‐Hydroxybutyrate	C01089	1.15 × 10^7^	2.85 × 10^6^	4.03	4.92E^−4^	
	Citramalate	C00815	4.86 × 10^6^	1.94 × 10^6^	2.51	3.65E^−3^	
	Kinic acid	C00296	3.37 × 10^6^	1.17 × 10^6^	2.89	7.96E^−3^	
	D‐Malic acid	C00497	3.33 × 10^7^	4.09 × 10^7^	0.81	1.82E^−1^	2↓
	4‐Guanidinobutyric acid	C01035	7.94 × 10^6^	1.13 × 10^7^	0.70	4.74E^−2^	
Sugars & derivates	D(+)‐Glucose	C00031	1.52 × 10^7^	1.03 × 10^7^	1.48	6.83E^−3^	3↑
	β‐D‐Glucose	C00221	1.51 × 10^7^	9.49 × 10^6^	1.59	1.37E^−2^	
	Glucose−1‐phosphate	C00103	5.25 × 10^6^	4.09 × 10^6^	1.28	2.04E^−2^	

**TABLE 2:** Differentially accumulated flavonoid, phenolic acids, and alkaloids in longan pulp between SX and CL

Class	Compounds	cpd_id	Abundance (peak area)		FC	FDR	Trend
			SX	CL			↑ ↓
Flavonoid	Kaempferol 3‐*O*‐robinobioside	–	2.34 × 10^7^	1.57 × 10^7^	1.49	9.62E^−4^	10↑
	Kaempferol 3‐*O*‐rutinoside	C21833	2.28 × 10^7^	1.46 × 10^7^	1.56	5.48E^−4^	
	Biquercetin	–	1.88 × 10^7^	1.02 × 10^7^	1.83	6.62E^−4^	
	Luteolin−7‐*O*‐rutin	–	1.41 × 10^7^	9.73 × 10^6^	1.45	1.06E^−2^	
	Rutin, quercetin 3‐rutinoside	C05625	4.08 × 10^6^	1.99 × 10^6^	2.05	4.73E^−3^	
	Luteolin 7‐*O*‐glucoside, Cynaroside	C03951	3.78 × 10^6^	1.63 × 10^5^	23.22	4.92E^−4^	
	6‐Hydroxykaempferol−7‐*O*‐glucoside	–	3.14 × 10^6^	1.87 × 10^6^	1.67	3.55E^−3^	
	6‐Hydroxykaempferol−3‐*O*‐glucoside	–	3.01 × 10^6^	1.62 × 10^6^	1.86	1.35E^−2^	
	Isorhamnetin 3‐*O*‐neohesperidoside	–	1.78 × 10^6^	6.61 × 10^4^	26.89	5.67E^−5^	
	Lonicerin	–	1.54 × 10^6^	1.61 × 10^5^	9.59	3.67E^−4^	
	3′‐*O*‐methylquercetin rutinoside	–	3.87 × 10^6^	5.48 × 10^6^	0.71	8.61E^−3^	10↓
	L‐Epicatechin	C09727	3.64 × 10^6^	6.05 × 10^6^	0.60	2.45E^−2^	
	Luteolin 7‐*O*‐glucoside (or 5‐*O*‐glucoside)	–	3.31 × 10^6^	5.87 × 10^6^	0.56	2.15E^−2^	
	Quercetin 7‐*O*‐β‐D‐glucoside	C12639	6.91 × 10^5^	3.69 × 10^6^	0.19	3.18E^−3^	
	Hyperin, quercetin 3‐galactoside	C10073	6.66 × 10^5^	3.66 × 10^6^	0.18	1.91E^−3^	
	Isoquercitrin, quercetin 3‐*O*‐glucoside	C05623	6.03 × 10^5^	3.23 × 10^6^	0.21	4.43E^−3^	
	Isoquercitrin isomer 1	–	4.44 × 10^5^	1.82 × 10^6^	0.24	4.59E^−3^	
	Spiraeoside, quercetin 4′‐*O*‐glucoside	–	4.28 × 10^5^	1.74 × 10^6^	0.25	6.65E^−3^	
	Hesperetin 5‐*O*‐glucoside	C16422	4.18 × 10^5^	1.64 × 10^6^	0.26	3.55E^−3^	
	Isoquercitrin isomer 2	–	3.73 × 10^5^	1.80 × 10^6^	0.21	1.95E^−3^	
Phenolic acids	β‐glucogallin	C01158	1.04 × 10^7^	7.86 × 10^6^	1.32	8.92E^−2^	2↑
	1‐*O*‐β‐D‐glucopyranosyl sinapate	C02919	3.36 × 10^6^	7.06 × 10^5^	4.75	3.18E^−3^	
	Terephthalic acid	C06337	5.91 × 10^6^	8.20 × 10^6^	0.72	6.38E^−3^	2↓
	3,4,5‐trimethoxyphenyl‐β‐D‐ glucopyranoside	–	2.04 × 10^6^	4.96 × 10^6^	0.41	1.03E^−3^	
Alkaloids	*N*‐Acetylputrescine	C02714	1.98 × 10^6^	8.45 × 10^5^	2.35	1.16E^−2^	1↑
	6‐Deoxyfagomine	–	8.77 × 10^6^	1.49 × 10^7^	0.59	5.91E^−4^	7↓
	*N*‐benzylmethylene isomethylamine	–	7.81 × 10^6^	3.47 × 10^7^	0.23	1.59E^−3^	
	bis(*N*,*N*‐diethylethanaminium)−2‐aceta‐mido−1,5‐anhydro−2‐deoxy−1‐[‐hydroxy(phospho‐nato) methyl]‐D‐glucitol	–	1.84 × 10^6^	6.43 × 10^6^	0.29	6.39E^−3^	
	Trigonelline	C01004	9.39 × 10^5^	3.75 × 10^6^	0.25	7.58E^−4^	
	3‐{(2‐aminoethoxy)(hydroxy)phosphoryl] oxy}−2‐hydroxypropyl palmitate	–	6.30 × 10^5^	4.31 × 10^6^	0.15	2.01E^−2^	
	3‐{[(2‐aminoethoxy)(hydroxy)phosphoryl] oxy}−2‐hydroxypropyl−9,12‐octadecenoate	–	5.82 × 10^5^	4.08 × 10^6^	0.14	1.46E^−2^	
	*N*‐Methyl nicotinate	–	1.25 × 10^5^	2.99 × 10^6^	0.04	2.06E^−3^	

**TABLE 3:** Differentially accumulated lipids, nucleotides, vitamins, and quinones in longan pulp between SX and CL

Class	Compounds	cpd_id	Abundance (peak area)		FC	FDR	Trend
			SX	CL			↑ ↓
Lipids	γ‐Linolenic acid	C06426	2.18 × 10^7^	1.74 × 10^7^	1.25	2.35E^−1^	2↑
	α‐Linolenic acid	C06427	2.16 × 10^7^	1.77 × 10^7^	1.22	2.64E^−1^	
	Palmitic acid	C00249	1.96 × 10^7^	2.97 × 10^7^	0.66	4.02E^−2^	18↓
	LysoPC(16:0)	C04102	9.85 × 10^6^	3.60 × 10^7^	0.27	1.06E^−2^	
	1‐Palmitoyl‐sn‐glycero−3‐phosphocholine; LysoPC(16:0)	C04102	8.11 × 10^6^	2.94 × 10^7^	0.28	1.06E^−2^	
	(E)‐Oleic acid; Elaidic acid	C01712	4.61 × 10^6^	6.05 × 10^6^	0.76	9.79E^−2^	
	LysoPC(18:3; 2n isomer)	–	4.40 × 10^6^	1.90 × 10^7^	0.23	1.37E^−2^	
	isoPC(18:2)	–	3.09 × 10^6^	6.87 × 10^6^	0.45	5.77E^−2^	
	LysoPC(18:3)	–	1.66 × 10^6^	5.60 × 10^6^	0.30	8.23E^−3^	
	1‐linoleoyl‐sn‐glycero−3‐phosphocholine; LysoPC(18:2)	C04100	1.57 × 10^6^	3.88 × 10^6^	0.40	4.56E^−2^	
	LysoPE 18:1 (2n isomer)	–	1.54 × 10^6^	6.74 × 10^6^	0.23	1.46E^−2^	
	1‐Linoleoylglycerophosphocholine; PC(18:2);	C04100	1.49 × 10^6^	4.04 × 10^6^	0.37	5.65E^−2^	
	LysoPC(16:0; 2n isomer)	–	1.45 × 10^6^	6.50 × 10^6^	0.22	1.38E^−2^	
	1‐acyl‐sn‐glycero−3‐phospho‐ ethanolamine; LysoPE 18:1	C04438	1.12 × 10^6^	5.04 × 10^6^	0.22	1.37E^−2^	
	1‐acyl‐sn‐glycero−3‐phosphocholine, C_9_H_20_NO_7_PR, R=C_17_H_28_); LysoPC(18:0)	C04230	1.02 × 10^6^	5.42 × 10^6^	0.19	9.56E^−3^	
	LysoPC(18:0)	–	9.48 × 10^5^	5.13 × 10^6^	0.18	1.09E^−2^	
	1‐Oleoyl‐sn‐glycero−3‐phosphocholine; LysoPC(18:1)	C03916	8.46 × 10^5^	3.36 × 10^6^	0.25	1.76E^−2^	
	lysophosphatidylcholine 16:1	–	8.27 × 10^5^	3.16 × 10^6^	0.26	2.31E^−2^	
	(1‐hexadecanoyl‐sn‐glycero−3‐phosphoethanolamine); LysoPE 16:0	–	5.59 × 10^5^	3.80 × 10^6^	0.15	1.38E^−2^	
	LysoPC(18:0)	–	2.79 × 10^5^	1.48 × 10^6^	0.18	1.37E^−2^	
Nucleotides & derivates	Guanosine	C00387	2.95 × 10^7^	1.77 × 10^7^	1.67	1.23E^−2^	2↑
	Cytidine	C00475	1.08 × 10^7^	8.32 × 10^6^	1.29	4.57E^−2^	
	AMP, Adenosine 5′‐monophosphate	C00020	6.84 × 10^6^	1.05 × 10^7^	0.65	1.06E^−2^	1↓
Tannins	Procyanidin B2	C17639	2.81 × 10^6^	6.36 × 10^6^	0.44	2.45E^−2^	3↓
	Procyanidin B4	C10238	2.58 × 10^6^	5.81 × 10^6^	0.44	1.38E^−2^	
	procyanidin B−3	–	1.25 × 10^6^	2.61 × 10^6^	0.47	1.71E^−2^	
Vitamins	Pyridoxine	C00314	2.29 × 10^7^	1.16 × 10^7^	1.98	4.92E^−4^	1↑
Quinones	Rhamnone−2‐*O*‐β‐D‐glucopyranoside	–	6.60 × 10^6^	9.10 × 10^6^	0.73	3.77E^−2^	1↓
Others	Senkyunolide M	–	8.09 × 10^6^	5.41 × 10^6^	1.50	1.90E^−1^	1↑
	propyl 2‐(trimethylammonio)ethyl phosphate	–	2.09 × 10^6^	8.90 × 10^6^	0.24	1.06E^−2^	2↓
	Oleoylethanolamine	C20792	7.68 × 10^5^	1.12 × 10^7^	0.07	4.34E^−4^	

Among the DAMs of secondary metabolites, flavonoids and lipids were the two largest classes. Ten flavonoids mainly including 4 glycosides of kaempferol, biquercetin, luteolin‐7‐*O*‐rutin, rutin, luteolin 7‐*O*‐glucoside, and isorhamnetin 3‐*O*‐neohesperidoside were upregulated in the SX pulp, while the other 10 flavonoid mainly containing 5 glycosides of quercetin, L‐epicatechin, Luteolin 5‐*O*‐glucoside, hesperetin 5‐*O*‐glucoside, isoquercitrin isomer 1 and 2 were downregulated in the pulp of SX (Table 2). Moreover, higher level of 2 DAMs of phenolic acids (β‐glucogallin and 1‐*O*‐β‐D‐glucopyranosyl sinapate) and 1 DAM of alkaloids (N‐acetyl putrescine) were observed in SX longan pulp but lower level of 2 DAMs of phenolic acids and 7 DAMs of alkaloids were found in SX longan pulp (Table 2).

Among the DAMs of lipids, only two DAMs (γ‐linolenic acid and α‐linolenic acid), which showed the highest abundance in SX longan pulp, were upregulated in SX, but the other 18 DAMs mainly including palmitic acid, oleic acid, LysoPC, and LysoPE were downregulated in SX. Among the 3 DAMs of nucleotides, guanosine, and cytidine were upregulated in SX, while adenosine 5′‐monophosphate were downregulated in SX. All of the 3 DAMs of tannins were downregulated in SX. The DAMs of vitamin (pyridoxine) and others (senkyunolide M) were upregulated while DAMs of quinones (rhamnone‐2‐*O*‐β‐D‐glucopyranoside) and others (propyl 2‐[trimethylammonio] ethyl phosphate and oleoylethanolamine) were downregulated in SX pulp (Table 3).

### 3.4 KEGG enrichment of DAMs and important metabolic pathways

KEGG enrichment analysis showed that 89 DAMs were significantly enriched (FDR ≦ 0.05) into 14 pathways including aminoacyl‐tRNA biosynthesis, ABC transporters, biosynthesis of alkaloids derived from ornithine, lysine & nicotinic acid, biosynthesis of alkaloids derived from histidine & purine, biosynthesis of plant hormones, arginine & proline metabolism, biosynthesis of phenylpropanoids, phenylalanine, tyrosine & tryptophan biosynthesis, glycolysis/gluconeogenesis, flavone and flavonol biosynthesis, glucosinolate biosynthesis, butanoate metabolism and starch & sucrose metabolism (Figure 3c). In further, the downregulated DAMs were significantly enriched (FDR ≦ 0.05) into 6 pathways including aminoacyl‐tRNA biosynthesis, glucosinolate biosynthesis, biosynthesis of alkaloids derived from histidine & purine, ABC transporters, biosynthesis of plant hormones and biosynthesis of phenylpropanoids (Figure 3d). The upregulated DAMs were significantly enriched (FDR ≦ 0.05) into 7 pathways including ABC transporters, biosynthesis of alkaloids derived from ornithine, lysine & nicotinic acid, aminoacyl‐tRNA biosynthesis, glycolysis/gluconeogenesis, arginine & proline metabolism, starch & sucrose metabolism, and biosynthesis of secondary metabolites. ABC transporters and aminoacyl‐tRNA biosynthesis were common pathways enriched in both of the up‐ and downregulated DAMs (Figure 3d).

### 3.5 Integrative analysis of DAMs in carbohydrate, amino acid and secondary metabolism

**FIGURE 4:**
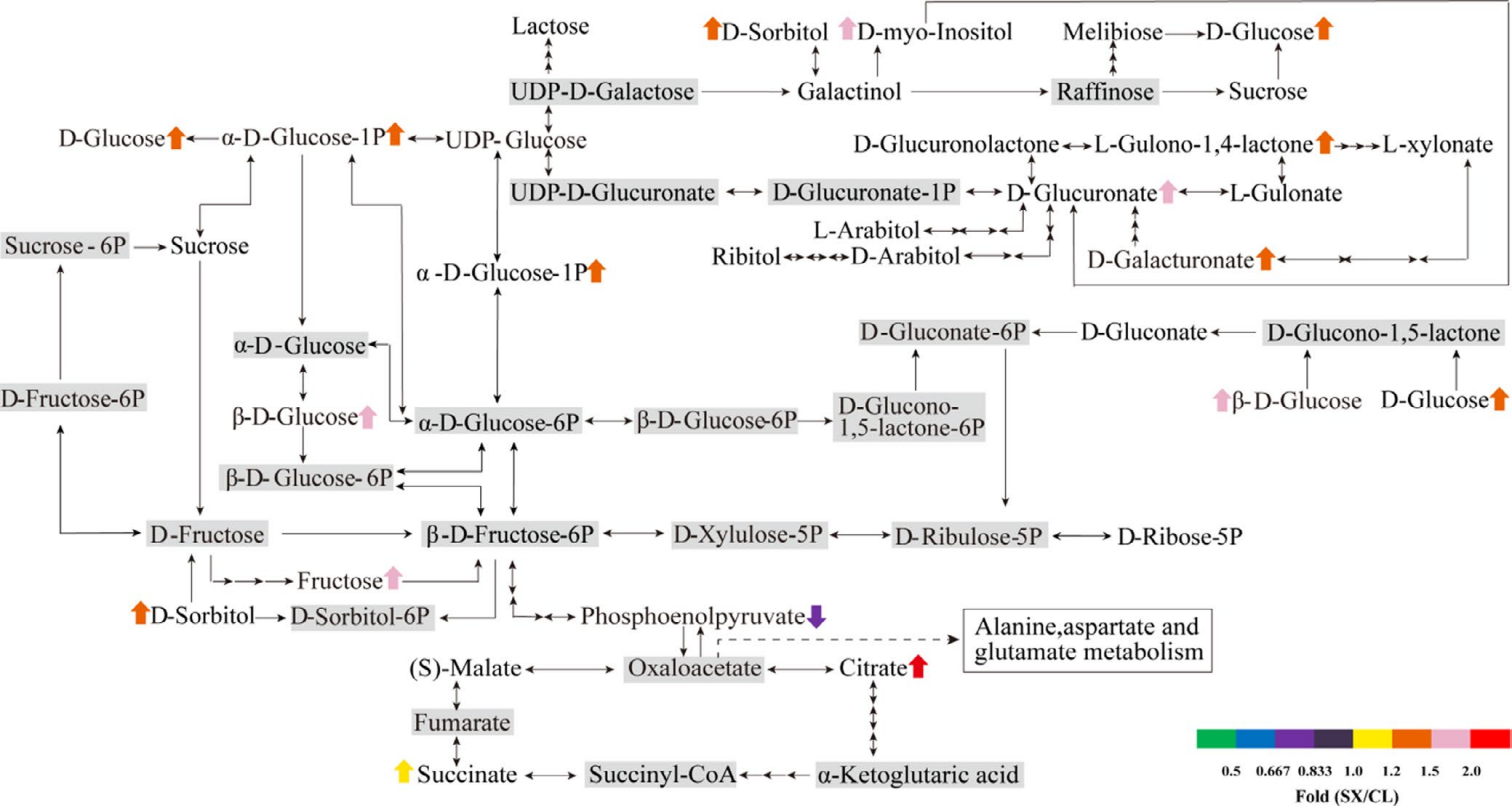
Differences in the content of sugar and organic acid between “Shixia” and “Chuliang” longan pulp (*p*‐value <.05, two paired *t*‐test). Note: upward arrow: upregulated; downward arrow: downregulated; gray background: undetected

Through a mapping of the sugars and acids, it was found that most of the monosaccharides (such as β‐D‐glucose, D [+]‐glucose, and fructose) and their derivatives (such as glucose‐1‐phosphate, α‐D‐glucose‐1P, β‐D‐glucose, galactinol, D‐myo‐inositol, D‐sorbitol) were upregulated in SX pulp. Disaccharides (such as sucrose, turanase, isomaltulose, lactose, D‐[+]‐trehalose, and melibiose) were detected, but were not significant different between SX and CL longan pulp (Figure 4). This result was consistent with the higher TSS and sweetness in SX when compared to CL longan (Figure 1a).

Seven sugar acids were also detected in both of the two longan pulp samples. Among them, L‐gulono‐1,4‐lactone, D‐glucuronate and D‐galacturonate showed higher accumulation (*p*‐value ≦.05) in SX pulp, but the content of D‐glucuronolactone, L‐xylonate, L‐gulonate, D‐gluconate showed no obvious difference between SX and CL (Figure 4). Moreover, 35 organic acids were detected in both of the two longan pulp samples. The accumulation of succinate, citrate, 3‐hydroxybutyrate, citramalate, kinic acid, (R)‐mevalonic acid, D‐galacturonic acid, and α‐hydroxy isobutyric acid were higher (*p*‐value ≦.05) in SX pulp, while that of 4‐guanidimobutyric acid, 2‐methyl succinic acid, phosphoenolpyruvate, phthalic acid, L‐homoserine, (S)‐(‐)2‐hydroxy isocaproic acid, and suberic acid were lower (*p*‐value ≦.05) in SX pulp. Among these organic acids, only three DAMs (phosphoenolpyruvate, citrate, and succinate), (S)‐malate and succinic acid were mapped into carbon metabolism pathways (Figure 4).

**FIGURE 5:**
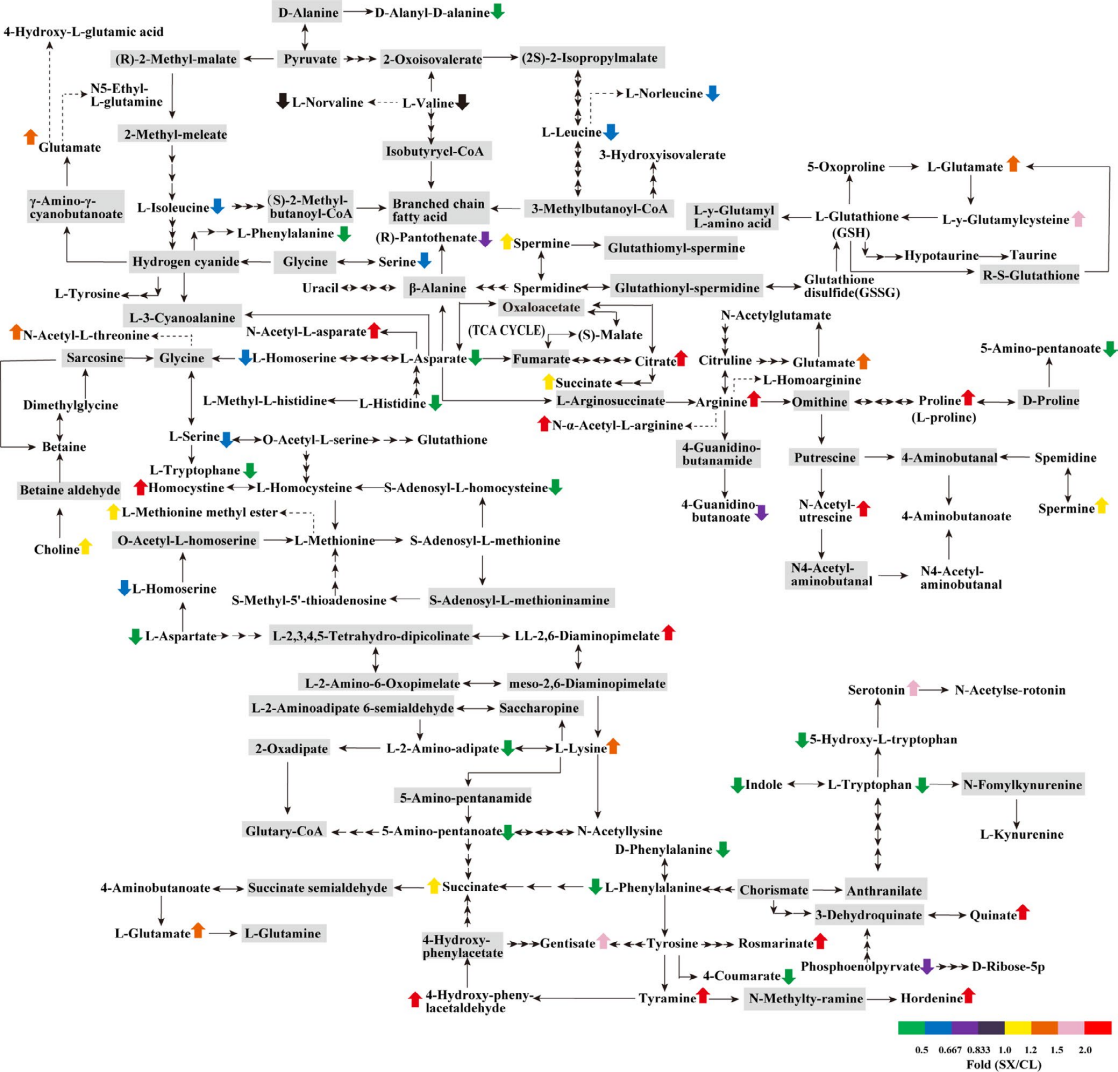
Difference in the content of amino acids between “Shixia” and “Chuliang” longan pulp (*p*‐value <.05, two paired *t*‐test). Note: upward arrow: upregulated; downward arrow: downregulated; gray background: undetected

Among the 70 detected amino acids, 7 essential amino acids (L‐lysine, L‐tryptophan, L‐phenylalanine, L‐methionine, L‐leucine, L‐isoleucine, and L‐valine) and 8 derivatives of them (N6‐acetyl‐L‐lysine, N‐acetyl‐D‐tryptophan, 5‐hydroxy‐L‐tryptophan, L‐2‐chlorophenylalanine, L‐methionine methyl ester, L‐norleucine, L‐norvaline, and N‐acetyl‐L‐threonine) were identified in SX and CL longan pulp. On the other hand, 8 nonessential amino acids (L‐histidine, L‐arginine, L‐proline, L‐aspartic acid, L‐serine, L‐glutamic acid, L‐citrulline and L‐tyrosine) and 13 derivatives of them (3‐methylhistidine, 1‐methylhistidine, N‐α‐acetyl‐L‐arginine, L‐Homoarginine, DL‐5‐oxoproline, N‐acetyl‐L‐aspartate, *O*‐acetylserine, L‐homoserine, N‐acetyl‐L‐glutamine, L‐theanine, 4‐hydroxy‐L‐glutamic acid, L‐homocitrulline and N‐acetyl‐L‐tyrosine) were identified. It was interesting to note that 10 dipeptides including (5‐L‐glutamyl)‐L‐amino acid, D‐alanyl‐D‐alanine, γ‐glu‐cys, DL‐alanyl‐DL‐leucine, phe‐phe, asp‐phe, L‐pyroglutamic acid, N‐glycyl‐L‐phenylalanine, N‐glycyl‐L‐leucine and N‐glycyl‐L‐isoleucine were detected in SX and CL longan pulp (Figure 5). In total, most of the essential amino acids except L‐lysine showed a lower accumulation in SX pulp, while except L‐tyrosine, three nonessential amino acids (L‐histidine, L‐aspartic acid, and L‐serine) were lower accumulated in SX pulp but the other four amino acids (L‐glutamic acid, L‐citrulline, L‐arginine, and L‐proline) showed higher accumulation in SX pulp (Figure 5).

**FIGURE 6:**
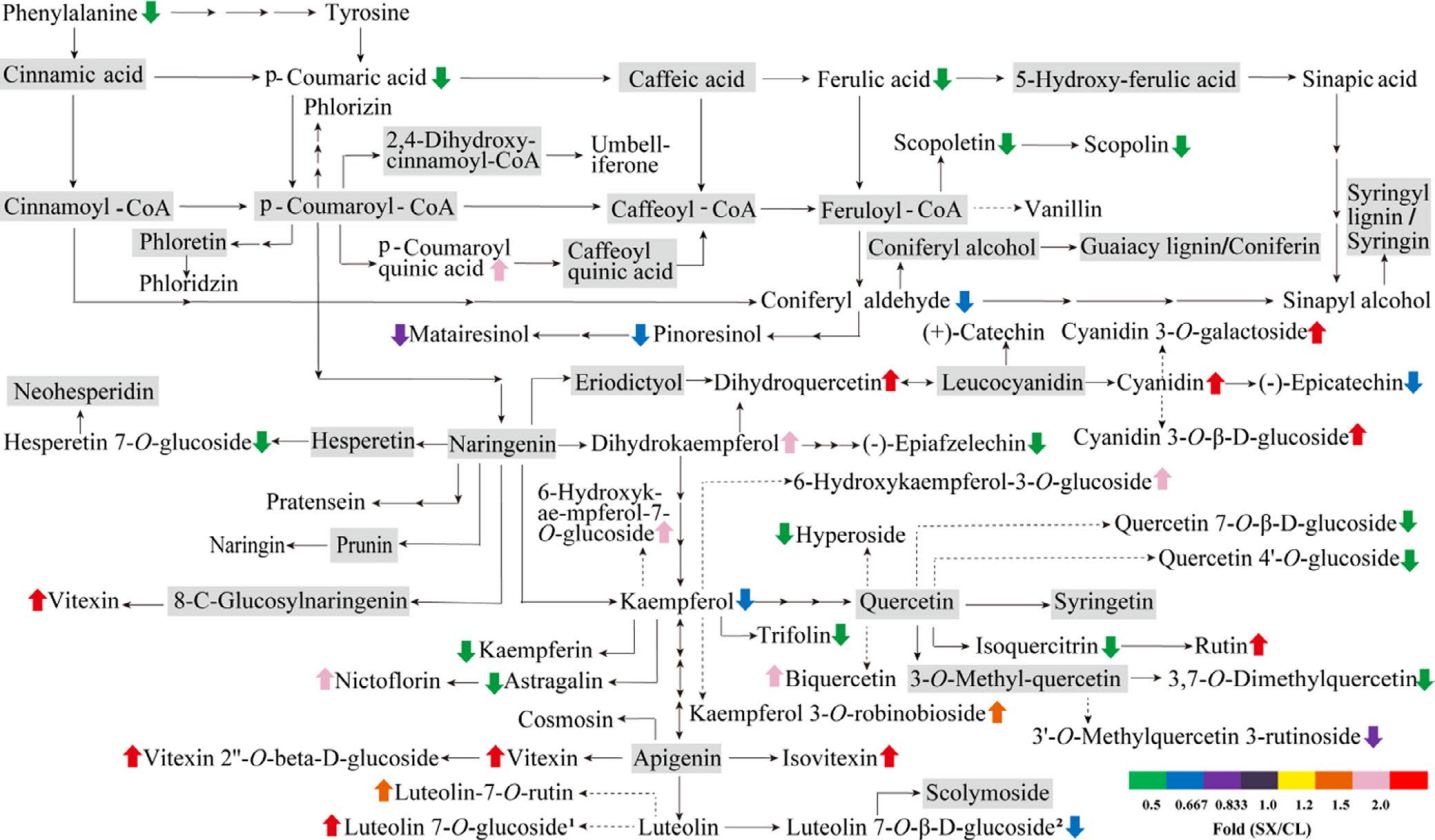
Differentially accumulated secondary metabolites between SX and CL longan pulp (*p*‐value <.05, two paired *t*‐test). Note: upward arrow: upregulated; downward arrow: downregulated; gray background: undetected

Longan fruits are rich in flavonoids and phenols which are mainly derived from phenylalanine ammonia‐lyase (PAL) synthesis. It was interesting to note that about 4‐folds downregulated accumulation of L‐phenylalanine was observed in SX longan pulp (Figure 6 and Table 1). Consistently, most of the phenolic acids from phenylpropanoid biosynthesis pathway except *p*‐coumaroyl quinic acid were downregulated in SX longan pulp. Flavonols such as kaempferol, kaempferol 3‐*O*‐α‐L‐rhamnoside (kaempferin), kaempferol 3‐*O*‐glucoside (astragalin), kaempferol‐3‐*O*‐galactoside (trifolin), as well as quercetin 3‐galactoside (hyperoside), quercetin 7‐*O*‐β‐D‐glucoside (gossypitrin), quercetin 4′‐*O*‐glucoside (spiraeoside), isoquercitrin (quercetin 3‐*O*‐glucoside) and 3,7‐Di‐*O*‐methylquercetin were found to be downregulated in SX longan pulp. However, dihydrokaempferol, 6‐hydroxykaempferol‐7‐*O*‐glucoside, 6‐hydroxykaempferol‐3‐*O*‐glucoside, nicotiflorin (kaempferol 3‐*O*‐rutinoside), kaempferol 3‐*O*‐robinobioside (biorobin), as well as dihydroquercetin, rutin (quercetin 3‐rutinoside), biquercetin, vitexin (apigenin 8‐C‐glucoside), isovitexin, vitexin‐2″‐*O*‐D‐glucopyranoside, luteolin‐7‐*O*‐rutin and luteolin‐7‐glucoside were upregulated accumulated in SX longan pulp. Thus, the composition of phenolic acids and flavonoids was significantly different between SX and CL longan pulp.

## 4 DISCUSSION

### 4.1 Metabolic basis of flavor‐related quality differences between SX and CL pulp

The ratio of sugar to acid performed a significant influence on fruit flavor (Li et al., 2014; Zheng et al., 2015). Previous reports indicated that the components and content of organic acids in longan pulp varied among varieties. More importantly, the content of organic acids in longan aril mainly including malic acid, oxaloacetic acid, α‐ketoglutaric acid, oxalic acid, citric acid, tartaric acid, and quinic acid was far lower than that of organic acids in other fruits such as citrus and apples (Hu et al., 2006). Our results showed that the accumulation of citric acid and quinic acid in SX was higher than that in CL, which was consistent with the experimental results of (Hu et al., 2006). A more comprehensive detection of organic acids, which were composed of 35 members, were presented in this study. Citric Acid, succinic acid, D‐malic acid, and citramalate, which were important intermediate metabolites in TCA cycle, showed the highest abundance in longan pulp and higher level in SX longan pulp. In addition, our previous results indicated that the content of the main sugars (sucrose, glucose, and fructose) in longan pulp were varied among varieties (Luo et al., 2018). It was worthy to note that the hexose and hexose‐phosphate such as β‐D‐glucose, D (+)‐glucose, glucose‐1‐phosphate, and glucose‐6‐phosphate showed the highest abundance among the monosaccharides in longan pulp and the content of these compounds was significant higher in SX longan pulp (Table 1 and Figure 4). These results may explained the difference of flavor between SX and CL longan fruits.

Amino acids were also the important contributors to flavor of fruits (Lu et al., 2017), which were abundant in longan fruit and greatly varied among cultivars (Dai et al., 2010). In total, 70 amino acids and derivatives were detected in SX and CL longan pulp. Dai et al. (2010) found that L‐glutamic acid showed the highest abundance in longan pulp. This result was consistent with our result. Dai et al. (2010) also found that all of the six essential amino acids (L‐threonine, L‐valine, L‐isoleucine, L‐leucine, L‐phenylalanine, and L‐lysine) together with three nonessential amino acids (L‐arginine, L‐glycine and L‐proline) showed higher accumulation in SX longan pulp. However, our results showed that most of the essential amino acids (L‐tryptophan, L‐phenylalanine, L‐methionine, L‐leucine, L‐isoleucine, and L‐valine) except L‐lysine showed a lower accumulation in SX pulp. Moreover, except L‐tyrosine, three nonessential amino acids (L‐histidine, L‐aspartic acid, and L‐serine) was lower accumulated in SX pulp but the other four ones (L‐glutamic acid, L‐citrulline, L‐arginine, and L‐proline) showed higher accumulation in SX pulp (Figure 5). These contrary results of essential amino acids (L‐phenylalanine, L‐leucine, L‐isoleucine and L‐valine) between our study and previous report indicated that strict control of preharvest operations and environment were required to make credible comparison of amino acids between two cultivars. In total, the difference of sugars, organic acids, and amino acids between SX and CL together acts as the metabolic basis for the difference of flavor between these two varieties.

### 4.2 Difference of secondary metabolites beneficial to human health between SX and CL pulp

Flavonoids and phenolic acids were the largest two groups of secondary metabolites in longan fruit. Recent studies have shown that polyphenols from longan seeds or flower performed benefits on human health, such as anti‐oxidation (Zheng et al., 2009), lowering cholesterol (Liu et al., 2012), lowering blood glucose (Yang et al., 2010), anti‐tumor (Li et al., 2018; Zhong et al., 2010), antibacterial and anti‐inflammatory (Lee et al., 2016). Our results showed that the content of many flavonoids and phenolic acids in pulp was significantly different between SX and CL longan.

Most of the metabolites in phenylpropanoid biosynthesis pathway (such as L‐phenylalanine, *p*‐coumaric acid, ferulic acid, scopoletin, scopolin, and coniferyl aldehyde) showed significantly lower accumulation but only *p*‐coumaroyl quinic acid showed higher accumulation in SX longan pulp (Figure 6). Two lignans (matairesinol and pinoresinol) derived from coniferyl aldehyde were also downregulated in SX pulp (Figure 6).

The flavonoid biosynthesis was linked with the phenylpropanoid biosynthesis pathway through the naringenin synthesized from *p*‐coumaroyl‐CoA by chalcone synthase (Chen et al., 2016; Li et al., 2019). The flavonoids including 97 members categorized as chalcones, dihydroflavonols, flavan 3‐ols, flavanones, flavones, flavone carbonosides, flavonols, and isoflavones were detected in SX and CL longan pulp. Among them, the flavanones such as hesperetin, prunin, eriodictyol, neohesperidin and their glucosides (except hesperetin 7‐*O*‐glucoside), which are abundant in citrus especially grapefruit and usually result in a primary bitter taste (Chen et al., 2016), were mostly not detected in SX and CL longan pulp (Figure 6). The representative polymethoxylatedflavones reported in Mandarin (*Citrus reticulata* Blanco) such as nobiletin, tangeretin, isosinensetin were also detected in SX and CL longan pulp. Moreover, the typical flavones reported in citrus such as diosmetin, diosmin, vitexin, aglycones of vitexin, isovitexin, luteolin, apigenin, chrysoeriol were also detected in SX and CL longan pulp. It was interesting to note that most of these flavones and their aglycones showed higher accumulation in SX longan pulp (Figure 6). Therefore, although the TPC and TFC were not significant different between SX and CL longan pulp, the great difference in the abundance of each secondary metabolites especially flavonoids and phenolic acids between SX and CL might lead to a distinct varied medicinal effect.

## 5 CONCLUSION

“Shixia” and “Chuliang” were the biggest two mainly‐planted longan cultivars in China. In this study, a systemic investigation of metabolic difference between SX and CL longan pulp was performed using an LC‐MS/MS based widely targeted metabolomics. Totally, 514 primary and secondary metabolites belonging to 23 categories were identified in the pulp of them, among which flavonoids were the most abundant, followed by amino acids, lipids and phenolic acids. A total of 89 metabolites with significant differential accumulation over 1.2 fold between “Shixia” and “Chuliang” were mainly enriched into pathways including flavone and flavonol biosynthesis, glycolysis/gluconeogenesis and arginine and proline metabolism. Higher accumulation of hexose and hexose‐phosphate (i.e., β‐D‐glucose, D[+]‐glucose, glucose‐1‐phosphate and glucose‐6‐phosphate), dominant organic acids (i.e., citric acid, succinic acid, D‐malic acid, and citramalate) as well as essential amino acids (L‐threonine, L‐valine, L‐isoleucine, L‐leucine, L‐phenylalanine and L‐lysine) in SX pulp might be contributed to the taste and flavor difference between SX and CL. Moreover, the great difference in content of secondary metabolites especially flavonoids and phenolic acids might result in different medicinal and nutritional characteristic between them. In conclusion, this study provided a systemic metabolic basis for understanding the nutritional differences between SX and CL. Further efforts are needed to deepen the molecular biology and pharmacology research on characteristic metabolites in longan pulp, a good resource for medicine and food.

## CONFLICT OF INTEREST

The authors declare that they have no conflicts of interest.

## ETHICAL APPROVAL

This study does not involve any human or animal testing.

## Data Availability

The data that support the findings of this study are available from the corresponding author, upon reasonable request.

## References

[fsn32552-bib-0001] Bonilla, E. , Akoh, C. C. , Sellappan, S. , & Krewer, G. (2003). Phenolics content and antioxidant capacity of Muscadine grapes. Journal of Agricultural and Food Chemistry, 51, 5497–5503. 10.1021/jf030113c 12926904

[fsn32552-bib-0002] Chen, J. J. , Peng, Z. X. , Shi, M. Y. , & Xuan, J. (2016). Advances in on flavonoid composition and metabolism in Citrus. Acta Horticulturae Sinica, 43, 384–400. 10.16420/j.issn.0513-353x.2015-0689

[fsn32552-bib-0003] Chen, W. , Gong, L. , Guo, Z. L. , Wang, W. S. , Zhang, H. Y. , Liu, X. Q. , Yu, S. B. , Xiong, L. Z. , & Luo, J. (2013). A novel integrated method for large‐scale detection, identification, and quantification of widely targeted metabolites: Application in the study of rice metabolomics. Molecular Plant, 6, 1769–1780. 10.1093/mp/sst080 23702596

[fsn32552-bib-0004] Chen, X. P. , Deng, C. J. , Hu, W. X. , Jiang, J. M. , Jiang, F. , Xu, Q. Z. , & Zheng, S. Q. (2015). Characteristics of soluble sugars in longan germplasm. Journal of Fruit Science, 32, 420–426. (in Chinese). 10.13925/j.cnki.gsxb.20140433

[fsn32552-bib-0005] Dai, H. F. , Huang, B. X. , Wang, X. R. , Li, J. G. , & Xiao, W. Q. (2010). Measurement of amino acids in flesh of 18 longan varieties by high performance liquid chromatography. Guangdong Agricultural Sciences, 37, 125–128. (in Chinese). 10.16768/j.issn.1004-874x.2010.10.035

[fsn32552-bib-0006] Eriksson, L. , Andersson, P. L. , Johansson, E. , & Tysklind, M. (2006). Megavariate analysis of environmental QSAR data. Part I−A basic framework founded on principal component analysis (PCA) partial least squares (PLS) and statistical molecular design (SMD). Molecular Diversity, 10, 169–186. 10.1007/s11030-006-9024-6 16770514

[fsn32552-bib-0007] Fraga, C. G. , Clowers, B. H. , Moore, R. J. , & Zink, E. M. (2010). Signature‐discovery approach for sample matching of a nerve‐agent precursor using liquid chromatography‐mass spectrometry, XCMS, and chemometrics. Analytical Chemistry, 82, 4165–4173. 10.1021/ac1003568 20405949

[fsn32552-bib-0008] Guo, X. M. , Luo, T. , Han, D. M. , & Wu, Z. X. (2019). Analysis of metabolomics associated with quality differences between room‐temperature‐ and low‐temperature‐stored litchi pulps. Food Science & Nutrition, 7, 3560–3569. 10.1002/fsn3.1208 31763006 PMC6848819

[fsn32552-bib-0009] Hu, Z. Q. , Li, J. G. , & Wang, H. C. (2006). Analysis of fruit sugar and acid compositions in the aril of different longan cultivars. Journal of Fruit Science, 23, 568–571. (in Chinese).

[fsn32552-bib-0010] Jia, Z. S. , Tang, M. C. , & Wu, J. M. (1999). The determination of flavonoid contents in mulberry and their scavenging effects on superoxide radicals. Food Chemistry, 64, 555–559. 10.1016/S0308-8146(98)00102-2

[fsn32552-bib-0011] Lee, C. H. , Chen, Y. S. , Hou, C. W. , Jeng, K. C. , & Chen, K. S. (2016). Anti‐inflammatory effect of longan seed extract in carrageenan stimulated sprague‐Dawley rats. Iranian Journal of Basic Medical Sciences, 19, 870–874. 10.22038/IJBMS.2016.7469 27746869 PMC5048123

[fsn32552-bib-0012] Li, D. D. , Luo, Z. S. , Mou, W. S. , Wang, Y. S. , Ying, T. J. , & Mao, L. C. (2014). ABA and UV‐C effects on quality, antioxidant capacity and anthocyanins content of strawberry fruit (*Fragaria ananassa* Duch.). Postharvest Biology and Technology, 90, 56–62. 10.1016/j.postharvbio.2013.12.006

[fsn32552-bib-0013] Li, D. , Zhang, X. C. , Li, L. , Aghdam, M. S. , Wei, X. X. , Liu, J. Q. , Xu, Y. Q. , & Luo, Z. S. (2019). Elevated CO_2_ delayed the chlorophyll degradation and anthocyanin accumulation in postharvest strawberry fruit. Food Chemistry, 285, 163–170. 10.1016/j.foodchem.2019.01.150 30797331

[fsn32552-bib-0014] Li, L. Y. , Xu, J. L. , Mu, Y. , Han, L. , Liu, R. H. , Cai, Y. P. , & Huang, X. S. (2015). Chemical characterization and anti‐hyperglycaemic effects of polyphenol enriched longan (*Dimocarpus longan* Lour.) pericarp tracts. Journal of Functional Foods, 13, 314–322. 10.1016/j.jff.2015.01.006

[fsn32552-bib-0015] Li, N. , Lin, Z. C. , Chen, W. , Zheng, Y. , Ming, Y. L. , Zheng, Z. Z. , Huang, W. , Chen, L. H. , Xiao, J. B. , & Lin, H. T. (2018). Corilagin from longan seed: Identification, quantification, and synergistic cytotoxicity on SKOv3ip and hey cells with ginsenoside Rh2 and 5‐fluorouracil. Food and Chemical Toxicology, 119, 133–140. 10.1016/j.fct.2018.05.018 29751073

[fsn32552-bib-0016] Liu, C. W. , Yang, D. J. , Chang, Y. Y. , Hsu, C. L. , Tseng, J. K. , Chang, M. H. , Wang, M. L. , & Chen, Y. C. (2012). Polyphenol‐rich longan (*Dimocarpus longan* lour.)‐flower‐water‐extract attenuates nonalcoholic fatty liver via decreasing lipid peroxidation and downregulating matrix metalloproteinases‐2 and ‐9. Food Research International, 45, 444–449. 10.1016/j.foodres.2011.11.007

[fsn32552-bib-0017] Liu, X. L. , Zhao, M. M. , Wang, J. S. , Yang, B. , & Jiang, Y. M. (2008). Antioxidant activity of methanolic extract of emblica fruit (*Phyllanthus emblica* L.) from six regions in china. Journal of Food Composition & Analysis, 21, 219–228. 10.1016/j.jfca.2007.10.001

[fsn32552-bib-0018] López‐Ibáñez, J. , Pazos, F. , & Chagoyen, M. (2016). MBROLE 2.0‐functional enrichment of chemical compounds. Nucleic Acids Research, 44, 201–204. 10.1093/nar/gkw253 PMC4987872 27084944

[fsn32552-bib-0019] Lu, H. Y. , Ban, Z. J. , Wang, K. D. , Li, D. , Li, D. D. , Poverenov, E. , Li, L. , & Luo, Z. S. (2017). Aroma volatiles, sensory and chemical attributes of strawberry (Fragaria × ananassa Duch.) achenes and receptacle. International Journal of Food Science and Technology, 52, 2614–2622. 10.1111/ijfs.13548

[fsn32552-bib-0020] Luo, T. , Shuai, L. , Liao, L. Y. , Li, J. , Duan, Z. H. , Guo, X. M. , Xue, X. Q. , Han, D. M. , & Wu, Z. X. (2018). Soluble acid invertases act as key factors influencing the sucrose/hexose ratio and sugar receding in longan pulp. Journal of Agricultural and Food Chemistry, 67, 352–363. 10.1021/acs.jafc.8b05243 30541284

[fsn32552-bib-0021] Paolo, B. , Saverio, O. , Mirko, M. , Matteo, B. , Lara, G. , & Azeddine, S.‐A. (2018). Gene expression and metabolite accumulation during strawberry (*Fragaria* × a*nanassa*) fruit development and ripening. Planta, 248, 1143–1157. 10.1007/s00425-018-2962-2 30066220

[fsn32552-bib-0022] Park, S. J. , Park, D. H. , Kim, D. H. , Lee, S. J. , Yoon, B. H. , Jung, W. Y. , Lee, K. T. , Cheong, J. H. , & Ryu, J. H. (2010). The memory‐enhancing effects of *Euphoria longan* fruit extract in mice. Journal of Ethnopharmacology, 128, 160–165. 10.1016/j.jep.2010.01.001 20064595

[fsn32552-bib-0023] Sheng, K. M. , & Wang, H. J. (2010). Advances in research of chemical constituents and pharmacological activites of *arillus longan* . Chinese Journal of Experimental Traditional Medical Formulae, 16, 236–238. (in Chinese). 10.3969/j.issn.1005-9903.2010.05.073

[fsn32552-bib-0024] Shuai, L. , Liu, H. , Liao, L. Y. , Lai, T. T. , Lai, Z. Y. , Du, X. X. , Duan, Z. H. , Wu, Z. X. , & Luo, T. (2021). Widely targeted metabolic analysis revealed the changed pigmentation and bioactive compounds in the ripening *Berchemia floribunda* (Wall.) Brongn. fruit. Food Science & Nutrition, 9, 1375–1387. 10.1002/fsn3.2093 33747452 PMC7958575

[fsn32552-bib-0025] Shuai, L. , Qian, P. H. , Liu, W. H. , Han, D. M. , & Wu, Z. X. (2016). Sugar contents and composition in the mature fruit of different longan cultivars. Chinese Journal of Tropical Crops, 37, 915–921. (in Chinese). 10.3969/j.issn.1000-2561.2016.05.011

[fsn32552-bib-0026] Tang, Y. Y. , He, X. M. , Sun, J. , Li, C. B. , Li, L. , Sheng, J. F. , Xin, M. , Li, Z. C. , Zheng, F. J. , Liu, G. M. , Li, J. M. , & Ling, D. N. (2019). Polyphenols and alkaloids in byproducts of longan fruits (*Dimocarpus longan* Lour.) and their bioactivities. Molecules, 24, 1186. 10.3390/molecules24061186 PMC6471414 30917573

[fsn32552-bib-0027] Wang, S. C. , Tu, H. , Wan, J. , Chen, W. , Liu, X. Q. , Luo, J. , Xu, J. , & Zhang, H. Y. (2016). Spatio‐temporal distribution and natural variation of metabolites in citrus fruits. Food Chemistry, 199, 8–17. 10.1016/j.foodchem.2015.11.113 26775938

[fsn32552-bib-0028] Wang, Z. R. , Cui, Y. Y. , Vainstein, A. , Chen, S. W. , & Ma, H. Q. (2017). Regulation of fig (*Ficus carica* L.) fruit color: Metabolomic and transcriptomic analyses of the flavonoid biosynthetic pathway. Frontiers in Plant Science, 8, 1990. 10.3389/fpls.2017.01990 29209349 PMC5701927

[fsn32552-bib-0029] Wu, Y. L. , Yi, G. J. , Zhou, B. R. , Zeng, J. W. , & Huang, Y. H. (2007). The advancement of research on litchi and longan germplasm resources in China. Scientia Horticulturae, 114, 143−150. 10.1016/j.scienta.2007.07.016

[fsn32552-bib-0030] Xiao, W. Q. , Lai, Z. Y. , Dai, H. F. , Huang, B. X. , Li, J. G. , Liu, C. H. , & Wang, X. R. (2007). Determination of nine nucleosides from Dimocarpus longan Lour. flesh by HPLC. Journal of Huazhong Agricultural University, 26, 722–726. (in Chinese). 10.13300/j.cnki.hnlkxb.2007.05.015

[fsn32552-bib-0031] Xu, J. D. , Yan, J. J. , Li, W. J. , Wang, Q. Y. , Wang, C. X. , Guo, J. X. , Geng, D. L. , Guan, Q. M. , & Ma, F. W. (2020). Integrative analyses of widely targeted metabolic profiling and transcriptome data reveals molecular insight into metabolomic variations during apple (*Malus domestica*) fruit development and ripening. International Journal of Molecular Science, 21, 4797. 10.3390/ijms21134797 PMC7370097 32645908

[fsn32552-bib-0032] Yang, B. , Jiang, Y. M. , Zhao, M. M. , Chen, F. , Wang, R. , Chen, Y. L. , & Zhang, D. D. (2009). Structural characterization of polysaccharides purified from longan (*Dimocarpus longan* Lour.) fruit pericarp. Food Chemistry, 115, 609–614. 10.1021/jf902534v

[fsn32552-bib-0033] Yang, C. X. , He, N. , Ling, X. P. , Ye, M. L. , Zhang, C. X. , Shao, W. Y. , Yao, C. Y. , Wang, Z. Y. , & Li, Q. B. (2008). The isolation and characterization of polysaccharides from longan pulp. Separation and Purification Technology, 63, 226–230. 10.1016/j.seppur.2008.05.004

[fsn32552-bib-0034] Yang, D. J. , Chang, Y. Y. , Hsu, C. L. , Liu, C. W. , Lin, Y. L. , Lin, Y. H. , Liu, K. C. , & Chen, Y. C. (2010). Antiobesity and hypolipidemic effects of polyphenol‐rich longan (*Dimocarpus longans* lour.) flower water extract in hypercaloric‐dietary rats. Journal of Agricultural and Food Chemistry, 58, 2020–2026. 10.1021/jf903355q 20088600

[fsn32552-bib-0035] Yi, Y. , Zhang, M. W. , Liao, S. T. , Zhang, R. F. , Deng, Y. Y. , Wei, Z. C. , Tang, X. J. , & Zhang, Y. (2012). Structural features and immunomodulatory activities of polysaccharides of longan pulp. Carbohydrate Polymers, 87, 636–643. 10.1016/j.carbpol.2011.08.034 34663015

[fsn32552-bib-0036] Zhang, R. F. , Khan, S. A. , Lin, Y. S. , Guo, D. L. , Pan, X. W. , Liu, L. , Wei, Z. C. , Zhang, Y. , Deng, Y. Y. , & Zhang, M. W. (2018). Phenolic profiles and cellular antioxidant activity of longan pulp of 24 representative chinese cultivars. International Journal of Food Properties, 21, 746–759. 10.1080/10942912.2018.1425705

[fsn32552-bib-0037] Zhang, X. F. , Guo, S. , Ho, C. T. , & Bai, N. S. (2020). Phytochemical constituents and biological activities of longan (*Dimocarpus longan* Lour.) fruit: A review. Food Science and Human Wellness, 9, 95–102. 10.1016/j.fshw.2020.03.001

[fsn32552-bib-0038] Zheng, G. M. , Xu, L. X. , Wu, P. , Xie, H. H. , Jiang, Y. M. , Chen, F. , & Wei, X. Y. (2009). Polyphenols from longan seeds and their radical‐scavenging activity. Food Chemistry, 116, 433–436. 10.1016/j.foodchem.2009.02.059

[fsn32552-bib-0039] Zheng, L. J. , Nie, J. Y. , & Yan, Z. (2015). Advances in research on sugars,organic acids and their effects on taste of fruits. Journal of Fruit Science, 32, 304–312. (in Chinese). 10.13925/jcnki.gsxb.20140271

[fsn32552-bib-0040] Zhong, K. , Wang, Q. , He, Y. , & He, X. H. (2010). Evaluation of radicals scavenging, immunity‐modulatory and antitumor activities of longan polysaccharides with ultrasonic extraction on in s180 tumor mice models. International Journal of Biological Macromolecules, 47, 356–360. 10.1016/j.ijbiomac.2010.05.022 20685359

[fsn32552-bib-0041] Zhu, G. T. , Wang, S. C. , Huang, Z. J. , Zhang, S. B. , Liao, Q. G. , Zhang, C. Z. , Lin, T. , Qin, M. , Peng, M. , Yang, C. K. , Cao, X. , Han, X. , Wang, X. X. , van der Knaap, E. , Zhang, Z. H. , Cui, X. , Klee, H. , Fernie, A. R. , Luo, J. , & Huang, S. W. (2018). Rewiring of the fruit metabolome in tomato breeding. Cell, 172, 249–261. 10.1016/j.cell.2017.12.019 29328914

[fsn32552-bib-0042] Zhu, Q. Q. , Jiang, Y. M. , Lin, S. , Wen, L. R. , Wu, D. , Zhao, M. M. , Chen, F. , Jia, Y. X. , & Yang, B. (2013). Structural identification of (1→6)‐α‐D‐glucan, a key responsible for the health benefits of longan, and evaluation of anticancer activity. Biomacromolecules, 14, 1999–2003. 10.1021/bm400349y 23617585

